# Oral contraceptive formulation and socio-cognitive performance: a short communication

**DOI:** 10.1177/20451253251386245

**Published:** 2025-11-01

**Authors:** Minhal Mussawar, Sneha Chenji, Christine Bueno, Jennifer L. Gordon

**Affiliations:** Department of Psychology, University of Regina, Regina, SK, Canada; Department of Psychology, University of Regina, Regina, SK, Canada; Department of Psychology, University of Regina, Regina, SK, Canada; Department of Psychology, University of Regina, 3737 Wascana Parkway, Regina, SK S4S 0A2, Canada

**Keywords:** cognition, emotion, mood, oestrogen, oral contraceptive, progestin

## Abstract

**Background:**

Oral contraceptives (OC) offer a range of ethinyl estradiol (EE) doses and progestin types, with evidence indicating marked differences in cognitive and emotional abilities in OC users. However, it remains unclear whether dose variations in EE (low vs high) and progestin androgenicity (androgenic vs anti-androgenic) are associated with variations in cognitive and emotional abilities.

**Objectives:**

Our study aimed to investigate the cognitive and emotional effects of various OC formulations.

**Design:**

Online between-subjects experimental design.

**Methods:**

Based on regular monophasic OC formulation use, 96 participants (26 ± 7 years) were recruited and categorised into one of four groups: low EE androgenic (*n* = 26), high EE androgenic (*n* = 24), low EE anti-androgenic (*n* = 21) and high EE anti-androgenic (*n* = 25). The Repeatable Battery for the Assessment of Neuropsychological Status, emotion recognition task, and the Positive and Negative Affect Schedule were administered. Visual analogue scales were also administered to assess rejection sensitivity before and after a social exclusion task (Cyberball task). Analysis of variance (2 × 2) models were used to compare cognitive and socio-emotional abilities between groups.

**Results:**

Anti-androgenic users demonstrated higher intensity ratings for emotional faces, and heightened feelings of insecurity after a social stressor. Overall positive and negative affect, as well as performance on objective cognitive tests, were similar across OC formulations.

**Conclusion:**

In OC users, OC formulations containing an anti-androgenic progestin were associated with greater perceived intensity of emotional faces as well as heightened rejection sensitivity. However, these subtle differences in task performance did not translate to differences in overall affect or cognitive performance.

## Plain language summary

Comparing the psychological effects of different types of birth control pills

Birth control pills, also known as oral contraceptives (OCs), contain different types and amounts of hormones. These hormones include ethinyl estradiol (EE), which is a form of estrogen, and progestins, which can have different effects on the body depending on whether they are androgenic (having male hormone-like effects) or anti-androgenic (blocking male hormone effects). Past research has shown that OCs can influence how people think and feel, but it is still unclear how different hormone levels and types in OCs affect these areas. This study looked at how different OC formulations may affect thinking skills (like memory and attention) and emotional responses. Ninety-six women who regularly used the same type of OC were divided into four groups based on whether their pill had a low or high dose of EE and whether it contained an androgenic or anti-androgenic progestin. The four groups were: low EE androgenic, high EE androgenic, low EE anti-androgenic, and high EE anti-androgenic. Participants completed tests that measured thinking abilities, recognized emotions in facial expressions, and reported their mood. They also completed a social rejection task to see how sensitive they were to being excluded. The study found that women taking OCs with anti-androgenic progestins rated emotional facial expressions as more intense and felt more insecure after being socially excluded. However, there were no major differences between the groups in general mood or in cognitive test scores. In conclusion, the type of progestin in OCs may slightly affect how women experience emotions and social interactions, but these changes do not appear to impact overall mood or thinking skills. This information may be helpful for people choosing an OC that best suits their emotional and cognitive needs.

## Introduction

Oral contraceptive (OC) use is highly prevalent, with four out of five reproductive-aged women in the United States reporting OC use at some point in their lives.^1^ Combined OCs contain both ethinyl estradiol (EE), a synthetic form of the endogenous ovarian hormone 17-β estradiol, and progestin, a synthetic analogue of the naturally occurring hormone progesterone. The dose of EE can differ between OCs, as can the type of progestin used, with some having stronger androgenic properties than others. These factors affect an OC’s side effect profile and associated risk for adverse events, such as risk of venous thrombosis, myocardial infarction and generalised oestrogenic side effects such as nausea, breast tenderness and acne.^2–4^ However, much less is known about the psychological effects of different OC formulations. Recent reviews of the cognitive and psychological effects of OCs called for additional research examining the moderating effect of progestin androgenicity on cognitive and socio-emotional outcomes.^5,6^

The current study, therefore, aimed to examine the relationship between progestin androgenicity and the performance of cognitive and socio-emotional outcomes. A secondary exploratory aim was to examine the interaction between progestin androgenicity and EE dose on these outcomes.

## Methods

### Participants

Ninety-six women were recruited from across Canada and the United States via Facebook advertisements. Eligibility criteria included the following: regular use of a monophasic combined OC (21- or 28-day cycle), ages between 18 and 42 years, fluency in verbal and written English, regular menstrual cycles (25–35 days), and not pregnant or breastfeeding. Participants taking any medication that influenced their mood and cognitive functioning were excluded, as were those with a current diagnosis of a mood, psychotic, or neurocognitive disorder.

OC formulations were grouped into androgenic or anti-androgenic progestin formulations, as well as low EE (10–20 mcg) or high EE (30–35 mcg) dose.^7^ A total of 50 androgenic OC users (26 low EE, 24 high EE) and 46 anti-androgenic OC users (21 low EE, 25 high EE) were included. Participants were compensated with a $50.00 Amazon e-gift card for full participation. Given the different conditions between EE dose and progestin type, making four separate groups, this study was considered a between-subjects observational study and the reporting of this study conforms to the Strengthening the Reporting of Observational Studies in Epidemiology statement.^8^

### Materials and measures

At the start of a Zoom-facilitated laboratory session (approximately 60 min), the research assistant reviewed the consent form with the participant, answered any questions, and had participants indicate their consent to participate by answering the question ‘Do you consent to participate in this study?’ in an online demographic survey, as approved by the University of Regina Research Ethics Board. The demographic survey assessed age, family income, marital status, education (years and degree), ethnicity, duration and reason for OC use. The tests outlined below were then administered on the second week of their active pill phase between 3 and 6 pm to reduce potential time-of-day effects. All cognitive and socio-emotional measures used have been previously validated for web-based administration.^9–12^

Repeatable battery for the assessment of neuropsychological status (RBANS)^9,13^ assessed immediate memory/ learning, visuospatial ability, language, attention, and delayed memory, having been used in previous studies examining cognitive differences between phases of the menstrual cycle and OC use.^14^ Performance on the RBANS was scored manually using the RBANS scoring booklet and converted into percentiles based on age and education.

Cyberball task^11^ assessed rejection sensitivity and socio-emotional cognition. Participants were told to use mental visualisation of tossing the ball between two people that they know; however, the two ‘players’ were just computers, programmed to exclude the participant by no longer tossing to them after two throws. This task was completed over Zoom, with the researcher sharing their screen and giving participants control of it. This was done to ensure that the participant completed the task correctly and to increase validity. The pre-to-post changes in self-reported stress, feelings of insecurity, and contentedness, rated on a visual analogue scale, were calculated.

Japanese and Caucasian Facial Expressions of Emotion^15^ and neutral faces were used to assess emotion recognition abilities. Participants were shown 28 images of Caucasian and Japanese males and females expressing different emotions, including happiness, sadness, surprise, disgust, anger, and fear. Each image was presented once, and participants were asked to select the emotion that best corresponded to the image, and then select the intensity rating (ranging from 1 to 4, 1 = not at all intense and 4 = very intense) that they felt matched the emotion, using their mouse and keyboard. Four photos were presented for each emotion. The percent accuracy and intensity ratings for each emotion were used to analyse emotion recognition abilities. A recognition bias score for misclassifying neutral, positive, or negative emotion was also computed. Similar to the Cyberball task, this was administered via Zoom with the researcher sharing their screen and giving control to the participant.

Positive and Negative Affect Schedule (PANAS-X)^16^ was used to assess current affect. A sum of positive and negative phrases endorsed was used to calculate the corresponding positive and negative affect, respectively.

To reduce the possibility of order effects, cognitive test administration was counterbalanced (see Supplemental Figure 1 for the order of test administration) and the visual analogue scale was used after each test to assess self-reported emotion during the testing session.

### Statistical analysis

IBM SPSS (v28) software (Armonk, NY, USA) was used to perform statistical analysis. Participant data were assessed for normality using the Shapiro-Wilk test. Outlier removal of cognitive and emotional tasks was performed prior to analysis (score ⩾ third quartile + 3*interquartile range OR ⩽ first quartile − 3*interquartile range). Group comparisons were performed using a 2 × 2 analysis of variance (ANOVA) model to test for main effects of progestin type (andro+ vs andro−). A 2 × 2 ANOVA examined the interaction between progestin type and EE dose (low vs high). To account for unequal variances in accurately recognised emotions, a linear mixed model with emotion intensity ratings as the dependent variable and OC progestin type and EE dose as independent variables was computed for each emotion. Note that emotion intensity was only included in the analysis when the categorisation of emotional faces was correct; thus, the degrees of freedom associated with each emotion vary somewhat. Statistical significance was set at *p* < 0.05. The Benjamini-Hochberg false discovery rate (FDR) method was used to adjust for multiple comparisons.^17^

### Power calculations

The target sample size for the current study was 128 to detect a medium effect of progestin type, *f* = 0.25. Since this target was not reached due to recruitment difficulties, power calculations were re-performed as sensitivity analysis using G*Power v3.1 (Aichach, Germany). These calculations revealed that we were powered to detect a main effect of progestin type *f* = 0.29, with 80% power (alpha = 0.05).

## Results

### Participant characteristics

Seven hundred and ninety individuals completed the online eligibility form; of those, 415 were found to be potentially eligible and provided additional information about the study. Reasons for ineligibility included the following: not being on an OC falling in the categories under investigation (*n* = 142), taking psychotropic medication (*n* = 118), previously having irregular menstrual cycles (*n* = 40), having a diagnosed mental illness or neurocognitive disorder (*n* = 24), currently in psychotherapy (*n* = 21), currently pregnant or nursing (*n* = 15), being outside of the eligible age range (*n* = 10), and not being fluent in English (*n* = 5). Of the 415 invited to participate, 96 participants were recruited into the study and attended the virtual testing session. By design, the distribution of the number of participants was comparable between groups: 26 low EE andro+, 24 high EE andro+, 21 low EE andro− and 25 high EE andro−. Overall, the mean participant age was 26.4 years (*SD* = 6.9), and the mean education was 15.6 years (*SD* = 2.4). Approximately 38% indicated some university level of education, 33% had a Bachelor’s degree, 19% had a Graduate degree, and 10% had a high school diploma. In considering participant ethnicity, 62.4% identified as Caucasian, 19.4% Asian, 6.5% Hispanic/Latino, 3.2% Black, 2.2% Aboriginal and 6.5% other ethnicities. The average family income was greater than $113,000, and 53.8% indicated that they were single (never married), 41.9% were cohabiting or married, and 4.3% were divorced or separated. The average time on the OC pill for participants was 3.9 years (*SD* = 4.2). These demographic variables did not differ by EE dose or progestin type (*p*FDR = n.s.). Details on the demographics per group and the number of participants for specific OC formulations are displayed in Supplemental Tables 1 and 2, respectively. Correlations between independent variables are displayed in Supplemental Table 3.

### Effects on cognitive outcomes

There were no significant differences in performance for the RBANS task (Supplemental Table 4).

### OC effects on emotion recognition

**Figure 1.**
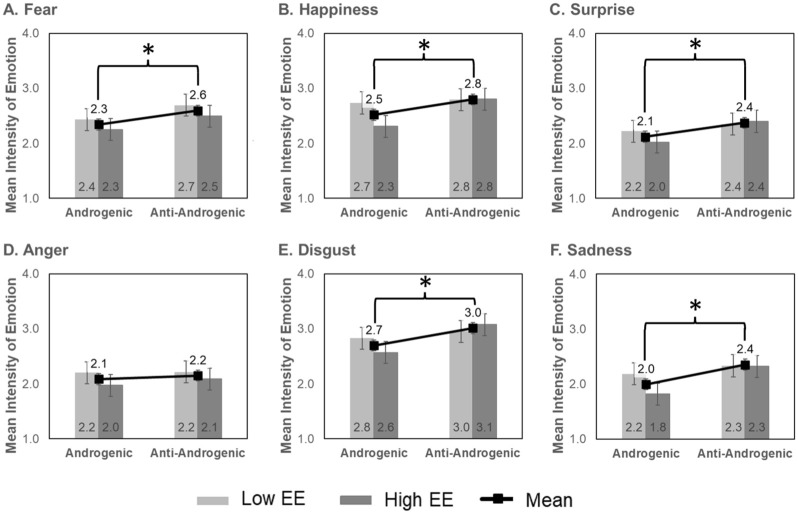
Mean intensity ratings of Zoom-facilitated emotion recognition task: (A) Fear, (B) Happiness, (C) Surprise, (D) Anger, (E) Disgust, and (F) Sadness. Bar graphs represent mean ratings for EE subgroups by progestin type. Line graphs (square markers) represent mean ratings for progestin groups. Error bars represent standard error. *Main effect of progestin, *p*FDR < 0.05. EE, ethinyl estradiol; FDR, false discovery rate.

Though percent accuracy in categorising emotional faces did not differ by progestin androgenicity or EE dose (Supplemental Table 5), a consistent main effect of progestin type was revealed for rating the intensity of emotions (Figure 1). Specifically, andro- groups had significantly higher emotional intensity ratings for fear, *F* (1, 150) = 4.45, *p*FDR = 0.037; happiness, *F* (1, 176) = 8.08, *p*FDR = 0.005; surprise, *F* (1, 104) = 5.32, *p*FDR = 0.023; disgust, *F* (1, 164) = 8.93, *pFDR* = 0.003; and sadness, *F* (1, 123) = 4.85, *p*FDR = 0.030, relative to andro+ groups.

**Figure 2.**
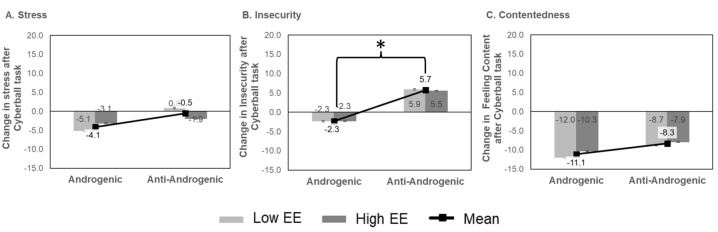
Mean change in self-reported emotional reactivity pre- and post-Cyberball task administered in a Zoom-facilitated session: (A) Stress, (B) Insecurity, and (C) Contentedness. Bar graph represents change in emotional reactivity as measured by the visual analogue scale for EE by progestin type. Line graph (square marker) represents the mean change in emotional reactivity for the progestin type. Error bars represent standard error. *pFDR < 0.05. EE, ethinyl estradiol; FDR, false discovery rate.

Analysis exploring reactivity to the Cyberball task revealed a significant main effect of progestin for feelings of insecurity, *F* (1, 80) = 7.70, *p*FDR = 0.007, η^2^_p_ = 0.08, where andro- groups reported greater increases in insecurity compared to andro+ groups (Figure 2). No differences in the other domains of emotional reactivity to the Cyberball task were noted.

No significant group differences were noted for endorsement of positive or negative emotions using the PANAS-X (Supplemental Table 5).

## Discussion

The current study found that, regardless of EE dose, progestin androgenicity was associated with emotion recognition. Specifically, the anti-androgenic group displayed higher emotion intensity ratings for fear, happiness, surprise, disgust, and sadness. This finding may explain research testing the effect of testosterone administration on emotion recognition in women. In these studies, a single dose of exogenous testosterone reduced women’s ability to identify emotions based on the eye region of facial expressions,^18^ the detection of anger in facial expressions,^19^ or trustworthiness in facial expressions.^20^ On an anatomical level, evidence suggests that androgenic hormones such as testosterone influence various aspects of amygdala circuitry in adolescents.^21^ Though minimal research has examined the effect of progesterone on amygdala circuitry, these findings may in part explain the increased emotional intensity ratings in the andro- group. However, more research is warranted.

Anti-androgenic progestin users also reported more intense feelings of insecurity following a social exclusion task, while androgenic progestin users reported a decrease in insecurity. This finding is consistent with research findings that higher testosterone levels in women were associated with lower rejection sensitivity, assessed via self-reported emotional responses to hypothetical scenarios involving interpersonal rejection.^22^ The idea that a more feminising hormonal regimen is associated with greater rejection sensitivity is also consistent with research showing that women exhibit a greater physiological stress response to social rejection challenges relative to men.^23^ However, the finding that OC formulation was unrelated to overall positive and negative affect suggests that these subtle effects on emotion recognition and rejection sensitivity do not necessarily translate into clinically significant effects on overall mood.

Neither EE dose nor progestin androgenicity was associated with cognitive performance. Though prior research has shown positive effects of oestrogen therapy on cognition in older women,^24^ it is perhaps unsurprising that the small EE dose differences seen across OC formulations would impact a sample of younger women. One review noted a positive association between progestin androgenicity and visuo-spatial ability, as well as a positive association between low oestrogenic activity, high progestin androgenicity and participants’ performance on mental rotation tasks and self-perceived masculinity.^5^ However, outside of visuospatial ability, minimal research has been published on both oestrogen dose and progestin type on cognitive performance. Our findings, though yielding no significant association between progestin androgenicity and cognition, add to current literature and challenge the existing hypothesis that cognitive performance may be impacted by progestin type and oestrogen dose.

### Limitations

Some notable limitations in the current study include its observational study design precluding any causal inferences, relatively small sample size, online test administration due to restrictions related to the COVID-19 pandemic – introducing variability through at-home distractions and technical disturbances – and the inclusion of only monophasic, single-cycle OCs, which limits generalisability to other OC types. Moreover, although the RBANS has been shown to be sufficiently sensitive to detect cognitive differences between OC users and non-users, it may be that various OC formulations cause more subtle differences than can be captured with the RBANS. Previous studies have demonstrated improved expressional fluency/language ability in monophasic OC users compared to triphasic users, but did not make any mention of progestin androgenicity.^25^ Had we included triphasic users, we may have noticed a similar pattern with language ability, possibly heightened by anti-androgenic progestin type. We may have also seen similar emotional intensity ratings and feelings of insecurity for anti-androgenic triphasic OC users. Future research should examine the effects of triphasic OCs versus monophasic OCs in more detail.

Despite these limitations, to the best of our knowledge, this is the first study to examine both EE dose and progestin androgenicity on cognitive and emotional parameters. Its confirmation of OC prescription via visualisation of the pill box over Zoom and its use of validated psychological measures and tasks are also strengths. Our findings are clinically relevant, suggesting that OC formulation is associated with small psychological effects that are detectable in a laboratory setting but have yet to be translated into clinical settings. This may be worth examining in future studies, given the medium effect size for feelings of insecurity following social rejection in anti-androgenic OC use, which could be of concern for some women starting the pill. There is a clear need for replication on a larger scale to further validate our findings. Nonetheless, for now, these findings may help reassure health-care providers that the selection of OC formulation can be based on patient acceptability and side effect profile.

## Conclusion

Our findings add to the current body of research, providing clarity that progestin androgenicity (but not EE dose) is associated with certain emotional abilities. OC groups did not differ on cognitive abilities. Future studies should consider examining other OC types alongside monophasic OCs to inform us further about socio-emotional or cognitive effects.

## Supplemental Material

sj-docx-1-tpp-10.1177_20451253251386245 – Supplemental material for Oral contraceptive formulation and socio-cognitive performance: a short communication Supplemental material, sj-docx-1-tpp-10.1177_20451253251386245 for Oral contraceptive formulation and socio-cognitive performance: a short communication by Minhal Mussawar, Sneha Chenji, Christine Bueno and Jennifer L. Gordon in Therapeutic Advances in Psychopharmacology

sj-docx-2-tpp-10.1177_20451253251386245 – Supplemental material for Oral contraceptive formulation and socio-cognitive performance: a short communication Supplemental material, sj-docx-2-tpp-10.1177_20451253251386245 for Oral contraceptive formulation and socio-cognitive performance: a short communication by Minhal Mussawar, Sneha Chenji, Christine Bueno and Jennifer L. Gordon in Therapeutic Advances in Psychopharmacology

## Declarations

**Ethics approval and consent to participate:** This study was approved by the University of Regina Research Ethics Board (#2020-085). As approved by the REB, informed consent was documented via an online survey administered at the start of the Zoom-facilitated laboratory session, prior to data collection.

**Consent for publication:** Not applicable.

**Availability of data and materials:** Data underlying the study cannot be made publicly available due to ethical concerns regarding participant confidentiality and consent, as imposed by the institutional ethics board. Data will be made available on request to qualified researchers under a confidentiality agreement. Researchers may send requests to Ara Steininger, the Research Compliance Officer at the University of Regina (research.ethics@uregina.ca).
