# Supplementary material for: Oral contraceptive formulation and socio-cognitive performance: a short communication
Source: Ther Adv Psychopharmacol. 2025 Nov 1;15:20451253251386245. doi: 10.1177/20451253251386245 (PMC12580508; doi:10.1177/20451253251386245)
Supplement: sj-docx-2-tpp-10.1177_20451253251386245 – Supplemental material for Oral contraceptive formulation and socio-cognitive performance: a short communication [file sj-docx-2-tpp-10.1177_20451253251386245.docx]

Supplemental Material


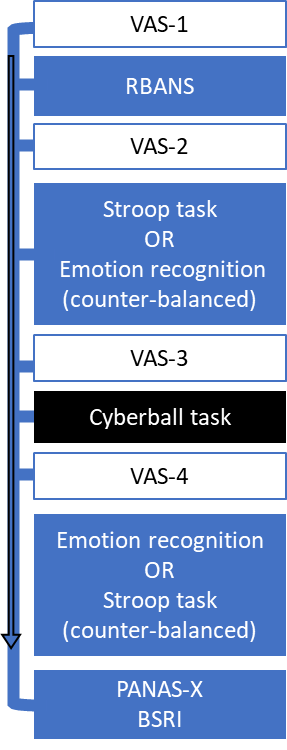


**Supplementary Figure 1.** Online laboratory session test administration and visual analogue scale (VAS) assessment timing. Abbreviations: VAS = Visual analogue scale, RBANS = Repeatable Battery for the Assessment of Neuropsychological Status, PANAS-X = Positive and Negative Affect Schedule, BSRI = Brief State Rumination Inventory.

**Supplementary Table 1.** Characteristics of participants (n = 96) across four OC formulation groups, recruited between September 2020 - May 2022.

|  | Overall | Andro+ | | Andro- | |
| --- | --- | --- | --- | --- | --- |
|  |  | Low EE | High EE | Low EE | High EE |
| N | 96 | 26 | 24 | 21 | 25 |
| Age (*M*, years)  (SD, min-max) | 26.4  (6.9, 18-42) | 23.3  (6.8, 18-42) | 28.6  (7.7, 20-42) | 27.5  (6.7, 18-40) | 26.7  (5.5, 19-39) |
| Average Family income (mode) | $113,000  and greater | $113,000 and greater | $113,000 and greater | $50,000 - $69,000 | $70,000 - $89,000 |
| Marital Status (%) |  |  |  |  |  |
| Single (Never married) | 53.8 % | 72.0 % | 45.8 % | 55.0 % | 41.7 % |
| Cohabiting / Married | 41.9 % | 28.0 % | 45.8 % | 45.0 % | 50.0 % |
| Divorced / Separated | 4.3 % | - | 8.3 % | - | 8.3 % |
| Education Level (%) |  |  |  |  |  |
| High School Diploma | 9.7 % | 4.0 % | 12.5 % | 20.0 % | 4.2 % |
| Some University | 37.6 % | 60.0 % | 20.8 % | 25.0 % | 41.7 % |
| Bachelor’s Degree | 33.3 % | 24.0 % | 37.5 % | 30.0 % | 41.7 % |
| Graduate Degree | 19.4 % | 12.0 % | 29.2 % | 25.0 % | 12.5 % |
| Education (*M*, years)  (SD, min-max) | 15.6  (2.4, 10-22) | 15.0  (2.5, 12-22) | 16.0  (2.3, 12-20) | 15.1  (2.5, 10-20) | 16.3  (2.0, 13-20) |
| Ethnicity (%) |  |  |  |  |  |
| Caucasian | 62.4 % | 68.0 % | 50.0 % | 65.0 % | 66.7 % |
| Asian | 19.4 % | 12.0 % | 37.5 % | 20.0 % | 8.3 % |
| Aboriginal | 2.2 % | 4.0 % | - | - | 4.2 % |
| Hispanic / Latino | 6.5 % | 4.0 % | 4.2 % | 10.0 % | 8.3 % |
| Black | 3.2 % | - | 8.3 % | 5.0 % | - |
| Other | 6.5 % | 12.0 % | - | - | 12.5 % |
| Average time on pill (*M*, years)  (SD, min-max) | 3.9  (4.2, 0.2-20) | 3.3  (4.2, 0.2-20) | 3.7  (4.3, 0.2-20) | 3.5  (4.1, 0.2-15) | 4.9  (4.3, 0.3-20) |

**Supplementary Table 2.** Oral contraceptive formulations sample size

| Pill formulations | N (%) |
| --- | --- |
| LE-andro+ | 26 |
| Levonorgestrel (0.10 mg) and ethinyl estradiol (20 mcg)  (e.g., Aviane, Alesse, Alysena, Lybrel) | 23 (88.5 %) |
| Norethindrone acetate (1 mg) and ethinyl estradiol (10 mcg)  (e.g., Loloestrin) | 2 (7.7 %) |
| Norethindrone acetate (1 mg) and ethinyl estradiol (20 mcg)  (e.g., Minastrin 24 Fe, Loestrin, Junel, Blisovi) | 1 (3.8 %) |
| HE-andro+ | 24 |
| Levonorgestrel (0.15 mg) and ethinyl estradiol (30 mcg)  (e.g., Altavera, Chateal, Marlissa, Seasonique) | 8 (33.3 %) |
| Norethindrone acetate (0.5 mg / 1.5 mg) and ethinyl estradiol (30 mcg / 35 mcg) (e.g., Loestrin 1.5/30, Junel 1.5/30, Brevicon, Ortho-Novum, Estarylla) | 14 (58.3 %) |
| Norgestrel (0.3 mg) and ethinyl estradiol (30 mcg)  (e.g., Ovral) | 2 (8.3 %) |
| LE-andro- | 21 |
| Drospirenone (3 mg) and ethinyl estradiol (20 mcg)  (e.g., Yaz, Nikki, Mya, Gianvi) | 21 (100 %) |
| Desogestrel (0.15 mg) and ethinyl estradiol (20 mcg)  (e.g., Mircette) | - |
| HE-andro- | 25 |
| Drospirenone (3mg) and ethinyl estradiol (30 mcg)  (e.g., Yasmin) | 9 (36.0 %) |
| Desogestrel (0.15 mg) and ethinyl estradiol (30 mcg)  (e.g., Marvelon, Mirvala, Apri) | 16 (64.0 %) |

Supplementary Table 3. Pearson correlations indicating associations between performance on cognitive and emotion tasks.

|  |  | **Demographics** | | | **RBANS** | | | | **Emotion Recognition - Accuracy** | | | | | | | **Emotion Recognition - Intensity** | | | | | | **PANAS** | | **Emotional Reactivity** | | | | |
| --- | --- | --- | --- | --- | --- | --- | --- | --- | --- | --- | --- | --- | --- | --- | --- | --- | --- | --- | --- | --- | --- | --- | --- | --- | --- | --- | --- | --- |
|  |  | Age | Education | OC duration | Immediate Memory | Language | Attention | Delayed  Memory | Neutral | Fear | Happiness | Surprise | Anger | Sadness | Disgust | Fear | Happiness | Surprise | Anger | Sadness | Disgust | Positive emotions | Negative emotions | Stress | Insecure | Rejection | Irritability | Content |
| **RBANS** | Immediate Memory | .13 | -.07 | .10 | -- |  |  |  |  |  |  |  |  |  |  |  |  |  |  |  |  |  |  |  |  |  |  |  |
|  | Language | .12 | .04 | .04 | **.35^**^** | -- |  |  |  |  |  |  |  |  |  |  |  |  |  |  |  |  |  |  |  |  |  |  |
|  | Attention | .15 | .08 | -.09 | .19 | **.25^*^** | -- |  |  |  |  |  |  |  |  |  |  |  |  |  |  |  |  |  |  |  |  |  |
|  | Delayed Memory | .08 | **.21*** | .07 | **.41^**^** | **.33^**^** | .13 | -- |  |  |  |  |  |  |  |  |  |  |  |  |  |  |  |  |  |  |  |  |
| **Emotion Recognition - Accuracy** | Neutral | .10 | **.24*** | -.10 | .07 | .15 | .03 | .12 | -- |  |  |  |  |  |  |  |  |  |  |  |  |  |  |  |  |  |  |  |
|  | Fear | -.12 | -.00 | -.13 | **.22^*^** | .15 | .01 | .14 | -.04 | -- |  |  |  |  |  |  |  |  |  |  |  |  |  |  |  |  |  |  |
|  | Happiness | .07 | .01 | .18 | .08 | **.27^*^** | -.04 | .07 | .14 | -.02 | -- |  |  |  |  |  |  |  |  |  |  |  |  |  |  |  |  |  |
|  | Surprise | -.12 | .06 | -.09 | .11 | .10 | .19 | .06 | .16 | .00 | .14 | -- |  |  |  |  |  |  |  |  |  |  |  |  |  |  |  |  |
|  | Anger | -.13 | -.19 | -.03 | .18 | .10 | .03 | **.27^**^** | .02 | **.23^*^** | -.02 | -.00 | -- |  |  |  |  |  |  |  |  |  |  |  |  |  |  |  |
|  | Sadness | -.02 | .09 | -.03 | .00 | .08 | -.01 | .16 | .16 | .12 | .15 | .09 | **.21^*^** | -- |  |  |  |  |  |  |  |  |  |  |  |  |  |  |
|  | Disgust | -.06 | -.16 | .07 | **.28^**^** | **.24^*^** | .07 | **.25^*^** | -.02 | .18 | .05 | .17 | .13 | -.02 | -- |  |  |  |  |  |  |  |  |  |  |  |  |  |
| **Emotion Recognition - Intensity** | Fear | -.19 | -.05 | -.13 | -.03 | -.04 | -.16 | .01 | -.20 | **.34^**^** | -.06 | -.19 | **.23^*^** | -.08 | -.02 | -- |  |  |  |  |  |  |  |  |  |  |  |  |
|  | Happiness | -.13 | -.08 | -.06 | .02 | -.11 | -.07 | -.14 | -.15 | .07 | **-.30^**^** | -.19 | .02 | -.08 | -.08 | .17 | -- |  |  |  |  |  |  |  |  |  |  |  |
|  | Surprise | .01 | .20 | .17 | **-.22^*^** | -.19 | -.16 | **-.29^**^** | -.01 | -.11 | -.10 | -.16 | -.07 | -.12 | **-.27^*^** | **.32^**^** | **.38^**^** | -- |  |  |  |  |  |  |  |  |  |  |
|  | Anger | .04 | .03 | -.06 | .01 | -.12 | -.03 | -.02 | -.04 | .10 | -.17 | -.08 | **.39^**^** | .06 | .10 | **.32^**^** | .10 | **.32^**^** | -- |  |  |  |  |  |  |  |  |  |
|  | Sadness | -.10 | -.04 | -.17 | .01 | .05 | .05 | -.28^**^ | .00 | **.23^*^** | -.10 | .11 | .05 | -.09 | -.08 | .19 | **.21^*^** | **.39^**^** | **.26^*^** | -- |  |  |  |  |  |  |  |  |
|  | Disgust | -.05 | -.08 | .09 | .04 | -.03 | -.10 | -.14 | -.09 | .08 | -.08 | -.00 | .05 | -.13 | -.11 | **.31^**^** | **.30^**^** | **.42^**^** | **.33^**^** | **.39^**^** | -- |  |  |  |  |  |  |  |
| **PANAS** | Positive emotions | **.33^**^** | .06 | **.25^*^** | .14 | -.11 | .07 | .08 | -.17 | .01 | -.05 | -.04 | .08 | -.02 | .09 | .09 | .05 | .10 | .12 | -.02 | .09 | -- |  |  |  |  |  |  |
|  | Negative emotions | .15 | .13 | .14 | -.19 | -.13 | -.11 | -.11 | -.13 | .00 | -.20 | **-.33^**^** | -.09 | -.06 | **-.23^*^** | .13 | .07 | **.28^**^** | .09 | .06 | .04 | .01 | -- |  |  |  |  |  |
| **Average Emotional Reactivity** | Stress | .01 | .07 | .06 | -.04 | .05 | .12 | -.01 | -.06 | .10 | .02 | .03 | -.04 | .01 | -.02 | .08 | .01 | .13 | .02 | .11 | .11 | -.11 | **.50^**^** | -- |  |  |  |  |
|  | Insecure | .19 | .15 | .01 | .02 | .01 | .07 | .10 | -.07 | -.06 | -.00 | -.03 | -.19 | -.01 | -.07 | -.10 | -.04 | .05 | -.10 | -.01 | .08 | -.10 | **.49^**^** | **.57^**^** | -- |  |  |  |
|  | Rejection | .11 | .18 | .03 | -.14 | -.10 | -.04 | .02 | .02 | -.07 | -.10 | -.11 | -.11 | .00 | **-.21^*^** | -.05 | .13 | .16 | -.07 | .02 | .00 | .02 | **.53^**^** | **.47^**^** | **.70^**^** | -- |  |  |
|  | Irritability | .12 | .13 | .19 | -.12 | -.15 | -.01 | -.06 | .07 | -.01 | -.14 | -.14 | -.07 | -.03 | **-.23^*^** | -.03 | .16 | .20 | .02 | .07 | .15 | -.02 | **.62^**^** | **.55^**^** | **.58^**^** | **.76^**^** | -- |  |
|  | Content | .02 | .01 | .02 | **.28^**^** | .17 | .09 | .09 | -.13 | .16 | -.17 | .03 | .02 | -.01 | .13 | .00 | .01 | -.14 | -.07 | .06 | -.18 | **.41^**^** | **-.31^**^** | **-.24^*^** | **-.21^*^** | -.14 | -.19 | -- |

** Correlation is significant at the 0.01 level (2-tailed).

* Correlation is significant at the 0.05 level (2-tailed).

**Supplementary Table 4.** Performance on Zoom-facilitated cognitive tasks. Scores are reported as mean percentile and standard error; age and education included as covariates.

| RBANS Domains | Andro+ | | Andro- | | Main effect  EE dose | | | Main effect  progestin | | | Interaction effect  EE dose x progestin | | |
| --- | --- | --- | --- | --- | --- | --- | --- | --- | --- | --- | --- | --- | --- |
|  | LE  *M* (SE) | HE  *M* (SE) | LE  *M* (SE) | HE  *M* (SE) | *F* | *p* | *η*^2^_p_ | *F* | *p* | *η*^2^_p_ | *F* | *p* | *η*^2^_p_ |
| Immediate Memory | 25.9 (5.1) | 30.4 (5.5) | 27.6 (6.0) | 28.8 (5.4) | 0.3 | .600 | .00 | 0.0 | .990 | .00 | 0.1 | .760 | .00 |
| Language | 47.2 (5.4) | 53.7 (5.7) | 45.7 (6.0) | 47.3 (5.7) | 0.5 | .482 | .01 | 0.5 | .494 | .01 | 0.2 | .671 | .00 |
| Attention | 10.0 (2.5) | 10.3 (2.8) | 8.3  (2.8) | 7.7  (2.7) | 0.0 | .961 | .00 | 0.6 | .433 | .01 | 0.0 | .874 | .00 |
| Delayed Memory | 33.8 (4.0) | 46.3 (4.2) | 34.2 (4.5) | 33.3 (4.2) | 1.9 | .171 | .02 | 2.2 | .143 | .03 | 2.5 | .117 | .03 |

RBANS = Repeatable Battery for the Assessment of Neuropsychological Status, RT = response time, ms = millisecond. *p*-values do not survive false discovery rate (FDR) corrections.

**Supplementary Table 5.** Performance on Zoom-facilitated emotion measures. Scores displayed as mean and standard error, age and education included as covariates.

|  | Andro+ | | Andro- | | Main effect  EE dose | | | | Main effect progestin | | | Interaction effect  EE dose x progestin | | |
| --- | --- | --- | --- | --- | --- | --- | --- | --- | --- | --- | --- | --- | --- | --- |
|  | LE  *M* (SE) | HE  *M* (SE) | LE  *M* (SE) | HE  *M* (SE) | *F* | *p* | | *η*^2^_p_ | *F* | *p* | *η*^2^_p_ | *F* | *p* | *η*^2^_p_ |
| Emotion Recognition Accuracy (%) | | | | | | | | | | | | | | |
| Neutral | 71.2 (4.5) | 83.7 (4.7)^a^ | 78.7 (5.1)^a^ | 69.8 (4.7)^b^ | 0.1 | .709 | | .00 | 0.4 | .511 | .01 | 5.0 | .027*^T^* | .05 |
| Fear | 48.1 (5.6) | 50.0 (6.0) | 40.0 (6.4) | 51.1 (5.9) | 1.2 | .278 | | .01 | 0.3 | .562 | .00 | 0.6 | .444 | .01 |
| Happiness | 95.8 (2.1) | 93.5 (2.1) | 95.8 (2.1) | 95.7 (2.1) | 0.3 | .558 | | .00 | 0.3 | .616 | .00 | 0.3 | .616 | .00 |
| Surprise | 64.4 (4.5) | 75.0 (4.7) | 67.5 (5.1) | 71.9 (4.7) | 2.4 | .122 | | .03 | 0.0 | .996 | .00 | 0.4 | .519 | .01 |
| Anger | 64.4 (5.7) | 62.0 (6.0) | 63.8 (6.5) | 63.5 (5.9) | 0.0 | .830 | | .00 | 0.0 | .945 | .00 | 0.0 | .857 | .00 |
| Disgust | 72.1 (5.6) | 79.3 (5.9) | 73.8 (6.4) | 67.7 (5.8) | 0.0 | .920 | | .00 | 0.7 | .402 | .01 | 1.2 | .267 | .01 |
| Sadness | 44.2 (4.5) | 52.2 (4.8) | 40.0 (5.2) | 48.1 (4.7) | 2.8 | .099 | | .03 | 0.7 | .393 | .01 | 0.0 | .985 | .00 |
|  |  |  |  |  |  | | |  |  | | |  | | |
| Recognition bias for neutral faces | | | | | | | | | | | | | | |
| Misclassified positive (%) | 15.6 (2.9) | 6.5  (2.9) | 10.0 (3.2) | 16.7 (2.9) | 0.2 | | .685 | .00 | 0.6 | .452 | .01 | 6.9 | **.010** | .07 |
| Misclassified negative (%) | 10.4 (3.8) | 9.8  (3.8) | 11.3  (4.2) | 13.5 (3.8) | 0.0 | | .833 | .00 | 0.3 | .559 | .00 | 0.1 | .709 | .00 |
|  |  |  |  |  |  | | | |  | | |  | | |
| Average Emotional Reactivity (VAS across 4 timepoints) | | | | | | | | | | | | | | |
| Stress | 32.6 (4.2) | 39.3 (4.5) | 29.9 (4.7) | 34.8 (4.4) | 1.7 | | .200 | .02 | 0.7 | .420 | .01 | 0.0 | .843 | .00 |
| Insecure | 18.4 (3.8) | 25.2 (4.1) | 16.7 (4.1) | 21.3 (3.7) | 2.0 | | .163 | .02 | 0.5 | .486 | .01 | 0.1 | .796 | .00 |
| Rejection | 9.7 (3.1) | 13.3 (3.3) | 9.6 (3.5) | 9.6 (3.2) | 0.3 | | .583 | .00 | 0.3 | .570 | .00 | 0.3 | .576 | .00 |
| Irritation | 18.1 (3.3) | 18.4 (3.5) | 15.8 (3.7) | 10.4 (3.4) | 0.5 | | .465 | .01 | 2.2 | .141 | .02 | 0.7 | .412 | .01 |
| Content | 56.3 (4.5) | 53.5 (4.8) | 41.8 (5.0) | 57.3 (4.7) | 1.8 | | .183 | .02 | 1.3 | .261 | .01 | 3.8 | .058 | .04 |
|  |  |  |  |  |  | | | |  | | |  | | |
| PANAS-X |  |  |  |  |  | | | |  | | |  | | |
| Positive Affect | 23.8 (1.4) | 25.4 (1.5) | 22.7 (1.5) | 27.1 (1.4) | 4.1 | | .046 | .04 | 0.5 | .825 | .00 | 0.9 | .337 | .01 |
| Negative Affect | 15.7 (1.0) | 14.3 (1.1) | 14.2 (1.1) | 13.7 (1.0) | .06 | | .454 | .01 | 0.5 | .490 | .01 | 0.0 | .840 | .00 |

VAS = visual analogue scale; PANAS-X = Positive and Negative Affect Schedule. Bold font indicates *p*-values that survive false discovery rate (FDR) corrections. *^T^* Strong trend. Post-hoc comparisons for accuracy in recognition of neutral faces: a > b (*p_FDR_* < .05).
